# Modeling and Optimization of Fractal Dimension in Wire Electrical Discharge Machining of EN 31 Steel Using the ANN-GA Approach

**DOI:** 10.3390/ma12030454

**Published:** 2019-02-01

**Authors:** Arkadeb Mukhopadhyay, Tapan Kumar Barman, Prasanta Sahoo, J. Paulo Davim

**Affiliations:** 1 Department of Mechanical Engineering, Jadavpur University, Kolkata 700032, India; arkadebjume@gmail.com (A.M.); tkbarman@gmail.com (T.K.B.); 2 Department of Mechanical Engineering, University of Aveiro, 3810-193 Aveiro, Portugal; pdavim@ua.pt

**Keywords:** WEDM, EN 31 steel, surface roughness, fractal dimension, ANN, GA

## Abstract

To achieve enhanced surface characteristics in wire electrical discharge machining (WEDM), the present work reports the use of an artificial neural network (ANN) combined with a genetic algorithm (GA) for the correlation and optimization of WEDM process parameters. The parameters considered are the discharge current, voltage, pulse-on time, and pulse-off time, while the response is fractal dimension. The usefulness of fractal dimension to characterize a machined surface lies in the fact that it is independent of the resolution of the instrument or length scales. Experiments were carried out based on a rotatable central composite design. A feed-forward ANN architecture trained using the Levenberg-Marquardt (L-M) back-propagation algorithm has been used to model the complex relationship between WEDM process parameters and fractal dimension. After several trials, 4-3-3-1 neural network architecture has been found to predict the fractal dimension with reasonable accuracy, having an overall R-value of 0.97. Furthermore, the genetic algorithm (GA) has been used to predict the optimal combination of machining parameters to achieve a higher fractal dimension. The predicted optimal condition is seen to be in close agreement with experimental results. Scanning electron micrography of the machined surface reveals that the combined ANN-GA method can significantly improve the surface texture produced from WEDM by reducing the formation of re-solidified globules.

## 1. Introduction

**Figure 1:**
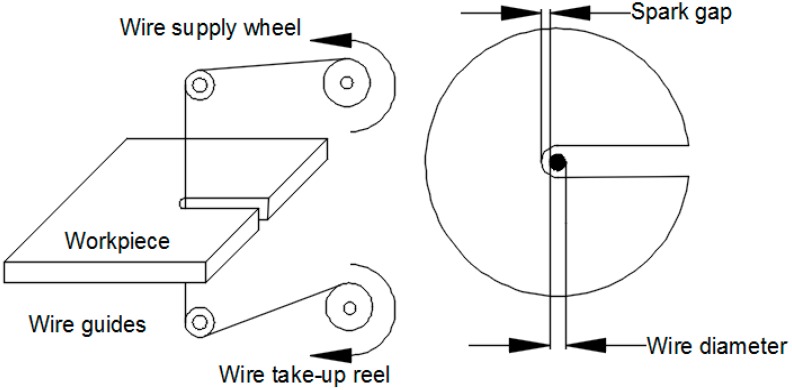
Schematic representation of the wire electrical discharge machining (WEDM) process.

A very popular non-conventional machining process which is capable of machining parts with intricate shapes and sharp edges is wire electrical discharge machining (WEDM). Though this process is widely used in tool and die making industries, its usage has been further extended to making micro-scale parts with high dimensional accuracy and surface finish [1,2]. The WEDM process has gained immense popularity for machining wear resistant and hard materials such as ceramics, nano-structured hardfacing alloys metal matrix composites, etc., with high machining accuracy [3,4,5]. WEDM utilizes a series of discrete sparks between the workpiece and tool electrode resulting in material erosion. The melted debris is flushed away by the dielectric medium (generally deionized water) [6]. The tool electrode in WEDM is a wire with a small diameter ranging between 0.05–0.25 mm. Since this is a non-contact type process, vibrations and chatter are prevented which introduce inaccuracies to machined parts [7]. However, wire breakage and bending are challenges that tend to limit the capabilities of this process, and a significant amount of research has been carried out to address this issue [8]. A schematic diagram of WEDM is shown in Figure 1.

From the tribological point of view, the quality of a machined surface, i.e., surface roughness, is an important parameter that affects the proper functioning of mating components in machines. Therefore, improvement of surface roughness and studies of the effect of controllable parameters on surface roughness have always been in focus. Such controllable parameters for WEDM include discharge current, open circuit voltage, wire speed, wire diameter, dielectric flushing pressure, pulse-on time, pulse-off time, spark gap, etc. [1,6,8]. The effect of WEDM process parameters and their significance in controlling the surface roughness has been widely studied through the use of design of experiments (DOE) and statistical tools [9,10,11,12]. The effect of pulse duration, open circuit voltage, wire speed and dielectric flushing pressure on the surface roughness of WEDM workpieces was investigated by Tosun et al. [9]. It was seen that surface roughness increases with an increase in pulse duration, open circuit voltage, or wire speed. Further, surface roughness improved with an increase in dielectric fluid pressure. The potential of WEDM for machining stainless clad steel in terms of surface roughness as a quality characteristic was investigated by Ishfaq et al. [7] using Taguchi’s orthogonal array (OA) and grey relational technique. Several parameters, such as the orientation of the workpiece (mild steel or stainless steel on top), the thickness of layers, wire diameter, pressure ratio, servo voltage, pulse-on time and wire feed rate were varied, and the surface roughness was observed. The highest significance in controlling the roughness was concluded for wire diameter. A larger wire diameter resulted in enhanced surface finish owing to a lower cutting speed and appropriate flushing of melted debris [7]. With the assistance of Taguchi’s quality design technique, a surface roughness of the order of 0.22 µm could be achieved by controlling process parameters such as machining voltage, current limiting resistance, type of pulse generating circuit, and capacitance [10]. In the case of a newly developed DC 53 die steel, pulse on time and pulse peak current were observed to have a significant effect on the surface roughness [11]. Pulse-on time, pulse-off time, and spark voltage have a major influence on the surface roughness and material removal rate (MRR) while machining high strength armor steel [12].

The influence of machining parameters on surface roughness and integrity has been also reported for a wide variety of materials of industrial importance, such as commercially pure titanium and its alloys, Inconel, multiwall carbon nanotube (MWCNT)-alumina composites, aluminum metal matrix composites, etc. [13,14,15,16,17]. The surface damage of WEDMed Ti-6Al-4V and Inconel 718 could be reduced by employing ultra-high frequency/short duration pulses and multiple trim passes [14]. This resulted in improved surface roughness and integrity with a low thickness recast layer which may be removed easily by etching. To improve the surface integrity of Nimonic 80A, Goswami and Kumar [15] suggested the setting of high pulse-off duration and low pulse-on duration. This again resulted in a lower recast layer thickness. Recast layer thickness and porosity in WEDM of MWCNT filled alumina composites may be reduced by adopting a multi-pass technique [16]. With each pass, the surface was smoothened by the removal of already-formed debris and cracks rather than removal of fresh material. The surface roughness and integrity may be also improved by carrying out post-processing such as grinding and etching-grinding to remove the recast layer [18]. This process was found to effectively reduce the surface roughness of Nimonic C 263 super alloy post-machining in WEDM [18].

Analysis of the surface and sub-surface layers formed after WEDM of Ti-6Al-4V alloy with and without heat treatment was reported by Mouralova et al. [19]. In both of the cases, a layer of molten metal stuck to the surface was observed. But for the heat-treated samples, this thickness was only 5–10 µm instead of 10–20 µm for the non-heat-treated samples. In fact, Goyal [20] reported the use of a cryogenically-treated zinc coated wire electrode to improve the machining performance and surface roughness for WEDM of Inconel 625 super alloy. Thus, even surface pre-treatments and wire electrode treatments also influence surface integrity.

Due to the stochastic nature of WEDM and too many adjustable machining parameters, it becomes rather difficult to utilize the machine optimally. Hence, in order to achieve efficient machining, modeling between input parameters and response variables is necessary. This may be carried out by adopting theoretical/empirical or artificial intelligence (AI) techniques. An efficient mathematical and statistical technique that has been employed in several research works is response surface methodology (RSM) [21]. Modeling and analysis of micro-WEDM of a titanium alloy were carried out by Sivaprakasam et al. [22]. A relationship between voltage, capacitance, and feed rate with MRR, kerf width, and surface roughness could be established with reasonable accuracy (coefficient of determination, R^2^ > 0.95). Finally, the genetic algorithm (GA) was employed for multi-objective optimization. A non-linear regression model [23], as well as a mathematical model using Buckingham’s pi theorem [24], were seen to have good modeling capabilities for surface roughness and WEDM process parameters.

Owing to simplified and unavoidable assumptions in mathematical models, AI-based techniques were seen to have better prediction capabilities. Such AI-based models include artificial neural networks (ANN), fuzzy logics, adaptive neuro-fuzzy inference systems (ANFIS), etc. Tzeng et al. [25] used RSM and a back-propagation neural network (BPNN) for analyzing the dependence of MRR and the surface roughness of pure tungsten on WEDM process parameters. There it was seen that the average prediction error was lower for the BPNN model in comparison with RSM based on regression models. Similarly, Saha et al. [26] observed overall mean prediction errors of 3.29% and 6.02% in BPNN and RSM approaches, respectively, for prediction of cutting speed and surface roughness in WEDM of WC-Co composites. Combining the modeling function of fuzzy inference and the learning ability of ANN led to the formation of an ANFIS model of WEDM with pulse duration, open circuit voltage, dielectric flushing pressure, and wire feed rate as input parameters [27]. The output parameters, namely white layer thickness and average surface roughness, could be predicted with reasonable accuracy using ANFIS architecture. Majumder and Maity [28] developed a general regression neural network (GRNN) architecture to model the surface roughness of nitinol with five critical WEDM parameters, namely pulse-on time, discharge current, wire feed, wire tension, and flushing pressure. The developed GRNN model had prediction capabilities with ±10% error.

From the existing literature, it may be observed that WEDM has gained immense importance due to its numerous advantages. Due to this, it has found its application in machining hard, fragile, and difficult to process materials in tool and die industries, as well as for generating complex surface geometries in mold walls [27,29]. WEDM also finds usage in aerospace, automobile, and nuclear industries since it provides an effective solution for machining materials with high hardness properties [25]. Several studies have been carried out to investigate the surface roughness and integrity of materials that find wide usage in industries (especially ceramics, titanium alloys, metal matrix composites, etc.) post-machining using WEDM [15,16,17]. The effect of WEDM process parameters and post-processing on surface integrity has been thoroughly investigated. Moreover, due to the stochastic nature of WEDM, mathematical as well as AI techniques have been employed to predict optimal machining parameters and to improve the surface roughness. However, the fractal dimension has been seldom used as a parameter to characterize surface roughness in WEDM [30,31,32]. The center line average surface roughness (R_a_) is dependent on parameters such as the resolution of the instrument used and the sampling length. To overcome this problem, the fractal dimension has been used in the present work to characterize surface roughness in WEDM. The effect of process parameters such as discharge current, voltage, pulse-on time, and pulse-off time on fractal dimension has been investigated. Furthermore, ANN has been used to model the complex relationship between fractal dimension and considered process parameters. Finally, the genetic algorithm (GA) is used to predict the optimal parametric combination using the developed ANN model.

## 2. Materials and Methods

The material on which WEDM was carried out is EN 31 tool steel. It is a high carbon steel with high hardness, compressive strength, and abrasion resistance [1]. Blocks with dimensions of 20 mm × 20 mm × 15 mm were selected as workpiece materials. The composition of EN 31 steel is 1.07% C, 0.57% Mn, 0.32% Si, 0.04% P, 0.03% S, 1.13% Cr, and 96.84% Fe. The modulus of elasticity, yield strength, ultimate tensile strength, and Poisson’s ratio of EN 31 steel are 197.37 GPa, 528.97 MPa, 615.40 MPa, and 0.294, respectively. Experiments were carried out on a five-axis CNC WEDM (Elektra, Maxicut 434, Kolkata, India). The wire electrode was zinc coated copper with a 0.25 mm diameter. The workpiece and electrode were separated by deionized water dielectric medium. The dielectric flow pressure was kept at 1.30 bar. The wire was fed at a rate of 8 m/min, and wire tension was 1000 gf. Discharge current, voltage, pulse-on time and pulse-off time were considered as the controllable/process parameters. They were varied at five levels, as shown in Table 1. A rotatable central composite design (CCD) was selected to reduce the number of experiments. Accordingly, 31 experiments were carried out with 16 factorial points, 8 axial points, and 7 center points. The roughness profiles of the machined workpieces were analyzed using a stylus-type profilometer (Talysurf, Taylor Hobson, Leicester, UK). A cutoff length, traverse speed, and traverse length of 0.8 mm, 1 mm/s and 4 mm were respectively selected. The obtained profile was then processed using Talyprofile software, and the fractal dimension was evaluated based on the structure-function method [33]. An average of 4 readings was considered. The sequence of experimental runs and the corresponding fractal dimension is outlined in Table 2. A scanning electron microscope (SEM) was used to observe the morphology of the machined surface at a near-optimum combination of parameters (JEOL, JSM 6360, Tokyo, Japan).

## 3. WEDM Process Modeling and Optimization Methodology Using ANN Integrated with GA

Since WEDM is a random and stochastic process, it is very difficult to map the input and output parameters. Neural networks have the flexibility of modeling input process parameters with output response variables without having prior knowledge of their relationship [26]. ANN is designed based on copying the human brain artificially [34]. ANN consists of a number of neurons organized in different layers. The neurons in different layers are connected by weights. The neural network architecture can be trained by adjusting the weights and other parameters. Several types of neural network architectures may be found in the literature [34]. In the present work, a feed-forward network with the Levenberg-Marquardt (L-M) back-propagation algorithm is used to train the network. It combines the Gauss-Newton algorithm with the steepest descent method to minimize the mean square error (MSE) of the output of the network. The feed-forward network consists of neurons that are grouped into the input, hidden, and output layers interconnected by weights. These weights are adjusted during the training stage of the learning process. The output *O*_*j*_ from a *j*^th^ neuron at any layer may be calculated as:

(1) $$O_{j}=f\sum_{i=1}^{n}w_{ij}x_{i}+b_{j}$$

where, *f* is the activation function (linear, logsigmoidal, tansigmoidal, etc.), *n* is the number of inputs to the *j*^th^ neuron or rather the number of neurons in the previous layer, *w_ij_* is the corresponding weight, *x_i_* is the output from *i*^th^ neuron, and *b*_*j*_ is the corresponding bias. 

**Figure 2:**
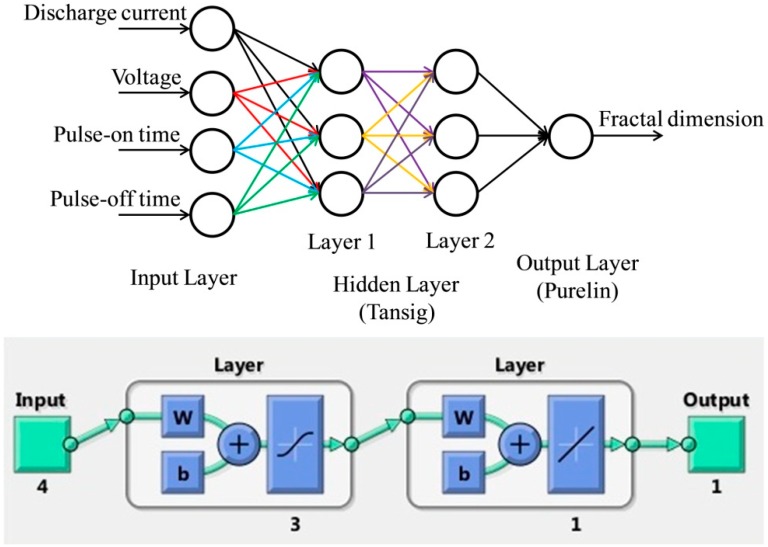
A 4-3-3-1 artificial neural network (ANN) architecture for the prediction of the fractal dimension.

In the present work, 2 hidden layers are considered with 3 neurons while 4 and 1 neurons are considered in the input and output layers respectively. Thus, this leads to a feed forward back-propagation network with 4-3-3-1 architecture. The activation function used between the input and hidden layers is “tansig” whereas a “linear” activation function is considered between the hidden and output layers. The aforesaid neural network architecture and the number of hidden neurons is selected carefully after several trial and error runs based on minimum MSE and higher correlation coefficient (R) of the model. Input parameters are normalized between −1 and 1. The results laid down in Table 2 are used to train, test, and validate the model. To train the network, 20 data is selected, while for testing, 6 data is chosen. The remaining 5 are used as validation sets. The ANN model may be represented as shown in Figure 2. MATLAB R2014b is used to develop the ANN model of WEDM.

On the other hand, GA has the capability to solve linear as well as non-linear stochastic problems by mimicking the principles of biological evolution. The essence of GA lies in the principle of “survival of the fittest”, wherein a population continuously evolves with iterations to achieve a better solution. Initially, the process begins with a set of potential solutions (in the form of bit strings) known as chromosomes. Each chromosome represents a set of process parameters. A population of possible solutions is created randomly within the search space from the chromosomes. The fitness of each of the chromosomes is determined from an objective function. After the fitness of the chromosomes is evaluated, then they evolve through biologically inspired processes in a generation or an iteration. In a generation, some individuals are selected based on their fitness value, which undergoes crossover and mutation. A pair of chromosomes exchanges genetic material in the crossover operation, while in mutation, changes are made to an individual chromosome. This enables the exploration of a broader search space. Thus, a new population is created and they again undergo the process of selection, reproduction, and evaluation in successive generations. This continues unless the global optimum solution is reached or the stopping criteria are met. 

In the present work, the ANN model of fractal dimension is used as an objective function for optimization using GA. The objective is to find out the optimal combination of discharge current, voltage, pulse-on time and pulse-off time to maximize the fractal dimension. A higher fractal dimension signifies a denser profile and a smoother topography [1]. This may be represented as:

Maximize: Fractal dimension (D)
Subject to: 2 Amp ≤ Discharge current ≤ 10 Amp
40 V ≤ Voltage ≤ 60 V
1 µs ≤ Pulse-on time ≤ 5 µs
1 µs ≤ Pulse-off time ≤ 5 µs

**Figure 3:**
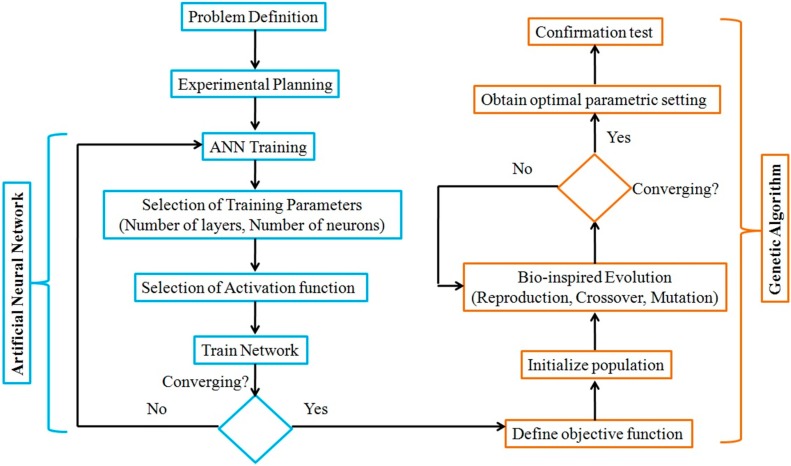
The integrated ANN-genetic algorithm (GA) approach for the modeling and prediction of fractal dimension in WEDM.

The neural network model of the process parameters and fractal dimension obtained is used as an input to the Genetic Algorithm Toolbox in MATLAB. Values of the fractal dimension are predicted by the ANN model for a given population size. In GA, there are no specific set of parameters mentioned in the literature that may yield the best results. But in the present work, the major parameters under consideration were population size, number of generations, crossover rate, and mutation rate. The population size considered is 50, and fitness scaling is done using a rank function. A uniform stochastic selection function is chosen for the determination of the parents for reproduction in the successive generation. The crossover function chosen is “scattered”, while 80% of the population size is selected for crossover. Lastly, mutation is carried out, and for this, an adaptive feasible mutation function is selected. This process continues for 500 generations unless the change in fitness function value is observed to be 10^−12^. The aforesaid GA parameters are considered based on the literature review [25] as well as the trial and error method. A schematic diagram of the combined ANN-GA approach is shown in Figure 3.

## 4. Results and Discussion

**Figure 4:**
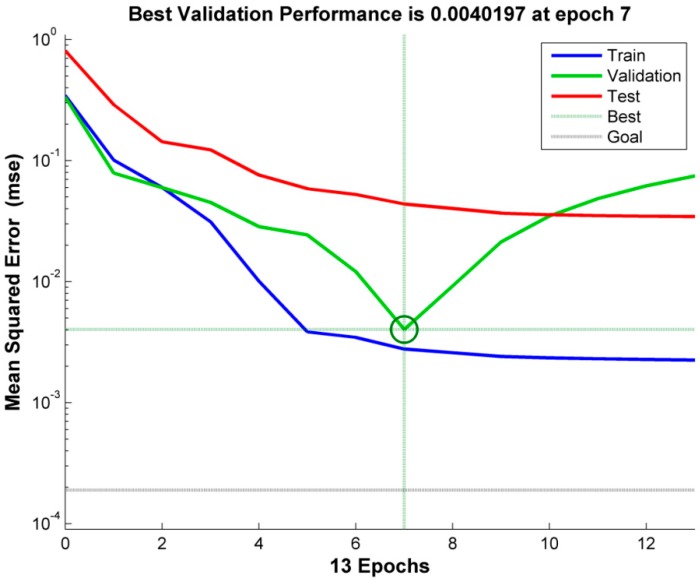
The lowest mean squared error for validation in 4-3-3-1 neural network architecture for the prediction of fractal dimension in WEDM.

**Figure 5:**
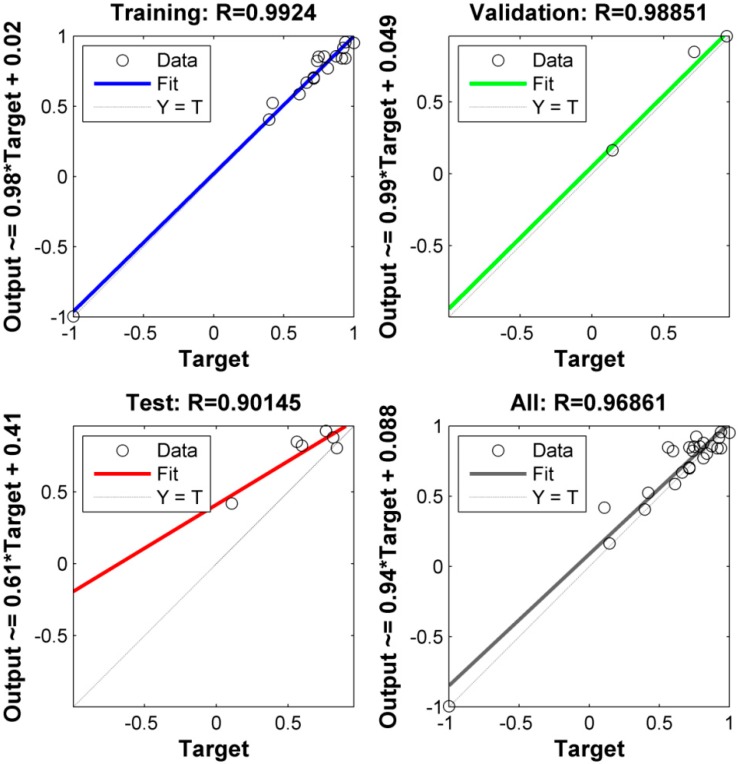
Training, testing, and validation performances of the 4-3-3-1 neural network architecture for the prediction of fractal dimension in WEDM.

The ANN model for the prediction of fractal dimension in WEDM of EN 31 is developed using MATLAB. A feed-forward network with an L-M training algorithm and 4-3-3-1 architecture with “tansig” activation function in hidden layers and “purelin” in the output layer gives the best correlation of WEDM process parameters with fractal dimension. The best architecture is also defined by the lowest MSE of the validation set. The best validation was observed at epoch 7 with an MSE of 0.0040197, as may be seen in Figure 4. The performance of the trained network may be also observed in Figure 5. The R-value of training is seen to be 0.99, which is significantly high and very near to 1. Moreover, the R-values of testing and validation are ~0.9 and ~0.99 respectively. For the overall prediction model, the R-value comes out to be ~0.97. Finally, the MSE values for training and testing patterns are 0.0001 and 0.0017, respectively. Recently, Yusoff et al. [35] employed a cascade forward back-propagation neural network (CFNN) for modelling of the multi-performances WEDM on Inconel 718. A 5-14-4 CFNN architecture could efficiently correlate machining parameters, namely pulse-on time, pulse-off time, peak current, servo voltage, and flushing pressure with material removal rate, R_a_, cutting speed, and sparking gap. An average error of 5.16% was generated with good agreement between predicted and experimental results. The results of the present work also indicate a high efficiency of ANN in modeling a non-linear and stochastic process like WEDM of EN 31 steel. The results obtained are also in accordance with the literature [26,35].

**Figure 6:**
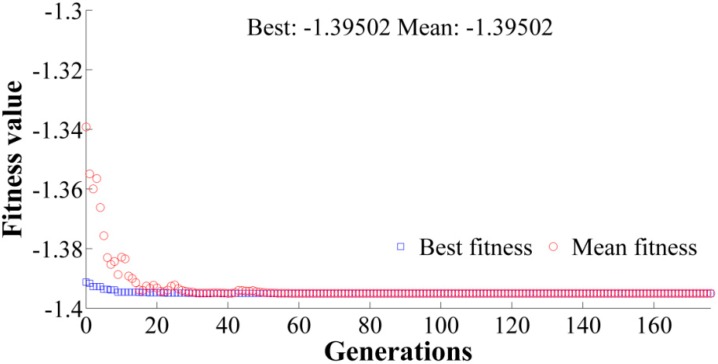
Variation of fractal dimension (fitness values) with generations.

The trained ANN model with high efficiency is used as the objective function for optimization using GA. The objective is to maximize the fractal dimension. Variation of the fitness value with the number of generations may be observed in Figure 6. After ~50 generations, a significant change in best fitness or mean fitness is not observed. The algorithm is hence terminated after 176 generations due to insignificant changes in the results in successive generations. Thus, the selection of GA parameters for optimization is justified. The optimal combination of machining parameters predicted by GA is 7.064 Amp discharge current, 60 V voltage, 3.215 µs pulse-on time, and 5 µs pulse-off time. The predicted value of the fractal dimension by the ANN-GA model is ~1.395. Exact values of the predicted process parameters could not be used for carrying out a confirmation test due to limitations with the WEDM setup. However, from Table 2, a combination of 6 Amp discharge current, 50 V voltage, 3 µs pulse-on time, and 5 µs pulse-off time results in a fractal dimension of 1.383 (Experiment 20) which is in close agreement with the predicted results. A higher value of fractal dimension was observed by Sahoo and Barman [1] with an increase in current and voltage for WEDM of EN 31 steel. Thus, the optimization results are in good agreement with their study. On the other hand, Das et al. [36] reported the use of an artificial bee colony (ABC) algorithm for the optimization of WEDM parameters considering the average surface roughness as the response. A parametric setting of 2 Amp discharge current, 60 V voltage, 5 μs pulse-on, time and 1 μs pulse-off time was predicted to yield a lower surface roughness of 2.244 µm. Thus, a clear difference in the predicted results may be observed for a scale-dependent roughness parameter and a scale-independent roughness parameter. From GA predicted results, it may be also seen that a lower pulse-on time and a higher pulse-off time is suggested for better surface finish. A higher pulse-on time causes a more powerful explosion and an increase in discharge energy and has a detrimental effect on surface roughness [25]. Similar observations have been also made for WEDM of pure tungsten, as well as armor materials [24,25]. Kumar et al. [17] observed an increase in R_a_ of aluminum metal matrix composites with an increase in pulse-on time, and a decrease in R_a_ was observed for an increase in pulse-off time. A higher pulse-off time results in an increased time gap between two successive sparks and hence it allows better solidifying and molten metal to be washed away from the cutting zone [37].

**Figure 7:**
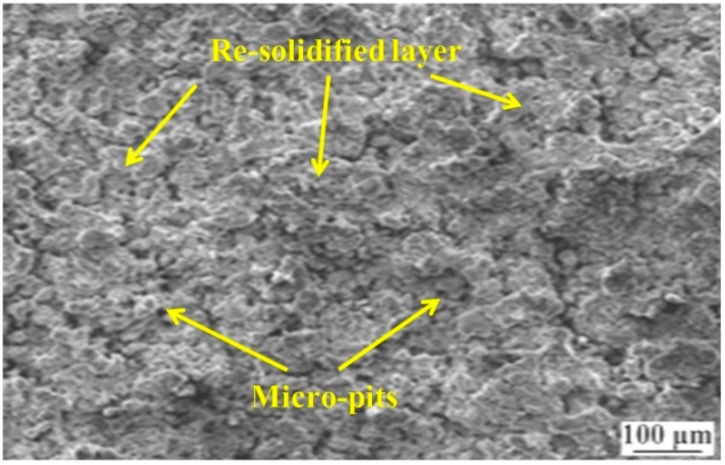
The machined surface corresponding to experiment number 25.

The SEM micrograph of a machined surface at 8 Amp current, 45 V voltage, 2 µs pulse-on time, and 4 µs pulse-off time is shown in Figure 7. This corresponds to the lowest value of fractal dimension (1.043) as can be observed in Table 2 (experiment 25). Pits are visible on the machined surface along with re-solidified particles of molten metal sticking onto each other. Consequently, the surface exhibits a high roughness. A high temperature is obtained in WEDM which causes the formation of molten metal during machining. Since it cannot flush away or vaporize all the molten metal, it re-solidifies to form lumps [28]. This re-solidified of molten metal thus forms a recast layer. In a recent study by Mandal et al. [18], grinding and etching have been proposed for the achievement of a surface finish of the order of 0.024 µm and complete removal of the recast layer. Such processes may be further adopted to improve surface integrity of EN 31 steel post-machining in WEDM. Also, higher current leads to higher discharge energy. This results in higher melting. Inadequate flushing/evaporation of molten metal may lead to a rough surface, as can be observed in Figure 7. Furthermore, trapped air bubbles may lead to the formation of micro-pits [28].

**Figure 8:**
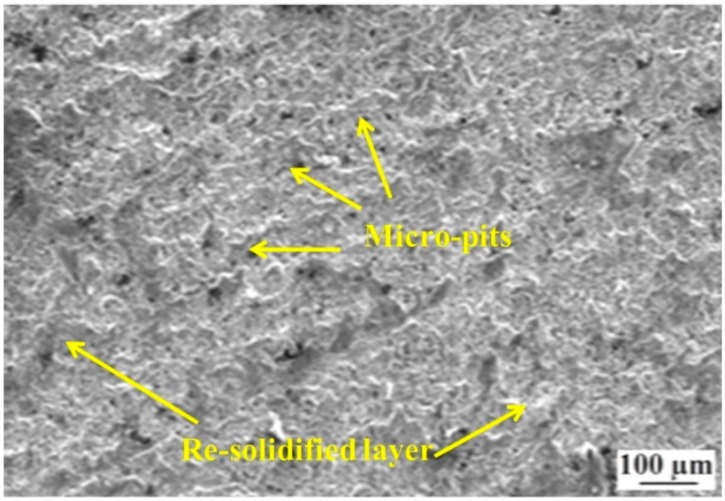
The machined surface corresponding to experiment number 20.

SEM micrography of a machined surface at a discharge current of 6 Amp, voltage of 50 V, pulse-on time of 3 µs, and pulse-off time of 5 µs is shown in Figure 8. This is the machined surface at a combination of parameters at near optimal conditions (experiment number 20 in Table 2) and here the surface appears to be quite smooth in comparison with Figure 7. Any cracks or craters may not be observed on the surface. The surface is also characterized by lower re-solidified globules due to the lower discharge current in comparison with Figure 7. This again establishes the fact that the relationship between WEDM process parameters and roughness has a complex nature, and GA has the capability of predicting optimal combinations with reasonable accuracy. In a recent study by Singh et al. [16], a multi-pass WEDM was seen to be capable of reducing the surface roughness drastically for MWCNT alumina composites in comparison with a single pass. Muthuramalingam et al. [38] observed that a diffused type of brass electrode produces better surface morphology in comparison with conventional brass wire or zinc coated electrodes. Reolon et al. [39] concluded that zinc coated copper wire produces better surface integrity for WEDMed Inconel alloy IN718 in comparison with uncoated brass wire. Thus, from the present analysis, the surface integrity of EN 31 could be improved without any post-processing or complexity while machining using WEDM. Nevertheless, such modifications may be included in future research works and prove to be beneficial in improving in fractal characteristics of EN 31 steel post-machining in WEDM.

## 5. Conclusions

In the present work, the fractal dimension is used to characterize the machined surface produced by WEDM on EN 31 steel. Four process parameters, namely discharge current, voltage, pulse-on time and pulse-off time, are varied at five equally-spaced levels. To reduce the number of experiments, a rotatable CCD experimental design methodology is adopted. The complex relationship between considered WEDM parameters and fractal dimension is modeled using ANN. After extensive modeling of the process, the following results may be directly inferred:

- A feed-forward network with an L-M training algorithm and 4-3-3-1 architecture is seen to have better prediction capability.
- The R-values during training, testing, and validation are 0.99, 0.9, and 0.99, respectively. The overall R-value of the model is 0.97.
- The MSE for training is 0.0001, and for the testing pattern it is 0.0017. This indicates the high efficiency of the formed ANN architecture in predicting fractal dimension with high accuracy. A good correlation between predicted and experimental results is therefore concluded.
- The predicted optimal combination of parameters from the combined ANN-GA is 7.064 Amp, 60 V, 3.215 µs, and 5 µs of discharge current, voltage, pulse-on time, and pulse-off time respectively. The predicted fractal dimension is 1.395.
- The SEM micrograph of the machined surface for the lowest value of the fractal dimension is characterized by re-solidified layers or recast layer and micro-pits.
- The SEM micrograph for a combination of parameters at a near-optimal combination is seen to be devoid of any pits or cracks. The re-solidified globules are lesser, indicating better vaporization and flushing. Thus, the integrated ANN-GA methodology is effective in optimizing the quality of a machined surface using WEDM.
- From the present model, a significant improvement in surface quality for WEDM of EN 31 steel could be achieved without any post-processing or pre-treatment, thereby optimizing time and cost of machining as well.

## Figures and Tables

**Table 1:** WEDM process parameters and their levels.

Controllable Factors	Unit	Levels				
		1	2	3	4	5
Discharge current	Amp	2	4	6	8	10
Voltage	Volt	40	45	50	55	60
Pulse-on time	µs	1	2	3	4	5
Pulse-off time	µs	1	2	3	4	5

**Table 2:** Combination of process parameters and experimental results.

Sl. No.	Discharge Current (Amp)	Voltage (V)	Pulse-On Time (µs)	Pulse-Off Time (µs)	Fractal Dimension
1	6	50	3	3	1.428
2	6	50	3	3	1.428
3	6	50	3	3	1.428
4	6	40	3	3	1.415
5	4	45	2	4	1.408
6	8	55	2	4	1.36
7	8	55	4	4	1.403
8	4	45	4	2	1.363
9	6	50	3	3	1.428
10	6	50	3	3	1.428
11	6	50	3	3	1.428
12	8	55	2	2	1.39
13	6	50	5	3	1.27
14	8	45	4	2	1.383
15	4	55	4	4	1.373
16	6	60	3	3	1.44
17	6	50	3	1	1.403
18	4	55	4	2	1.263
19	4	55	2	4	1.398
20	6	50	3	5	1.383
21	6	50	3	3	1.428
22	8	55	4	2	1.325
23	8	45	2	2	1.428
24	4	45	2	2	1.353
25	8	45	2	4	1.043
26	6	50	1	3	1.423
27	10	50	3	3	1.393
28	8	45	4	4	1.32
29	4	55	2	2	1.383
30	4	45	4	4	1.388
31	2	50	3	3	1.425
